# Effect of hearing aids on the externalization of everyday sounds

**DOI:** 10.1121/10.0028381

**Published:** 2024-09-03

**Authors:** Elin Roverud, Virginia Best

**Affiliations:** Department of Speech, Language and Hearing Sciences, Boston University, Boston, Massachusetts 02215, USA erover@bu.edu, ginbest@bu.edu

## Abstract

This study examined the influence of stimulus properties on sound externalization when listening with hearing aids. Normally hearing listeners were presented with broadband “tokens” (environmental sounds and speech) from loudspeakers, and rated externalization using a continuous scale. In separate blocks, they listened unaided or while wearing behind-the-ear hearing aids with closed domes and low gain (linear or compressive). There was a significant influence of token on ratings, even for unaided listening, and the effect of hearing aids depended on token. An acoustic analysis indicated that hearing aids were more likely to disrupt externalization for peakier sounds with a low-frequency emphasis.

## 1. Introduction

The primary function of hearing aids (HAs) is to improve the audibility of speech and other sounds for individuals with hearing impairment. Because HAs interrupt the natural sound path to the ear canals, however, they have the potential to distort spatial cues that listeners rely on for sound localization. Indeed, a number of studies have documented disruptions to sound localization for HA wearers (Noble and Gatehouse, 2006; Akeroyd and Whitmer, 2016), and in a recent study, we showed that listening through HAs can reduce the perceived distance of sounds and even lead to breakdowns of externalization (whereby sound images are perceived at or inside the head instead of out in the world; Best and Roverud, 2024). Disruptions to externalization were most apparent for HAs configured with behind-the-ear (BTE) microphones and occluding domes. While that study examined only speech stimuli, informal interviews with participants who were HA users indicated that certain non-speech sounds were most likely to be internalized in daily life (e.g., dishes clanging, doors slamming, keys jingling, trucks driving by). These reports are consistent with interview data collected by Boyd (2014), in which HA wearers noted internalized perceptions for loud, broadband, and impulsive sounds (e.g., doors banging), as well as for narrow bandwidth high-frequency sounds (e.g., alarms, doorbells). These anecdotal reports suggest that the effects of HAs on externalization may be more extreme for non-speech than speech sounds, and may depend on the acoustic properties of the sounds. The primary goal of the present study was to provide some behavioral data on this issue.

A secondary aim of the study was to examine whether linear vs compressive HA amplification would be more likely to degrade externalization. HAs programmed with wide-dynamic range compression systematically apply lower gain for higher input levels, with momentary gain determined by attack and release times (Dillon, 2012). Given that sound level is an especially important cue for auditory distance perception (Zahorik *et al.*, 2005), and that interaural level differences are critical for sound localization (Hartmann, 2021), it is often assumed that the nonlinear effects of compression may lead to altered spatial perception. However, the limited available data on this issue are somewhat equivocal. Akeroyd (2010) measured relative distance perception in HA wearers with their own HAs and found no evidence that performance depended on compression parameters. On the other hand, Wiggins and Seeber (2012) reported that compression adversely affected various spatial attributes of sounds (including externalization) for normally hearing listeners. Our previous study (Best and Roverud, 2024) did not examine this issue systematically but found breakdowns of externalization both for normally hearing listeners given linear gain and for hearing-impaired listeners given compressive gain. In the current study, we directly compared linear and compressive HAs in the context of externalization. Moreover, since compression depends strongly on the temporal characteristics of the input signal (Stone and Moore, 1992), we were interested to know whether differences between linear and compressive HAs would vary with sound type.

## 2. Methods

### 2.1 Participants

In total, 12 young adults with normal hearing (mean age, 24 years; range, 18–34 years) participated. All participants had audiometric thresholds <= 20 dB HL bilaterally at octave frequencies from 250 to 8000 Hz. Seven listeners participated in Experiment 1 and five participated in Experiment 2. Participants were college students or young professionals recruited via flyers and job postings in the Boston University community. They provided informed consent and were paid for their participation.

### 2.2 Hearing aids and fitting procedures

HAs were GN ReSound One receiver-in-the canal devices with Microphone and Receiver In-The-Ear (M&RIE, Hopkins, MN) technology. These HAs have 14 compression channels and a frequency range from 100 to 9550 Hz in an ear simulator according to manufacturer specifications. Based on our previous study (Best and Roverud, 2024), the devices were fit so as to have the largest impact on externalization, which meant programming the aids to use the BTE microphones on the body of the aids and coupling the aids to the ear canal using GN ReSound silicone instant ear tips of the strongly occluding power dome style.

Two pairs of HAs were programmed using ReSound SmartFit software. Only mild feedback cancellation was turned on, and all noise reduction and other special features were turned off. One pair of HAs (“linear”) was programmed to have 10 dB of linear gain (compression ratio = 1), and a maximum output level ranging from 97 to 107 dB and from 250 to 8000 Hz. This condition matched the “BTE Closed” condition described in Best and Roverud (2024). The other pair of HAs (“compressive”) was programmed to have 10 dB of gain for input levels up to and including 50 dB sound pressure level (SPL), but decreasing gain for input levels above this with a compression ratio of 1.5 from 250 to 8000 Hz. Syllabic time constants were used: 12-ms attack times and 70-ms release times (except for 250 and 500 Hz, where release times were 120 ms), based on manufacturer settings. The feedback manager calibration was run for each participant and HA pair prior to testing.

### 2.3 Equipment

Participants were seated in a single-walled sound-attenuating booth with perforated metal walls/ceiling and a carpeted floor (Industrial Acoustics Company, Hampshire, UK). The reverberation characteristics of this room are described elsewhere (Kidd *et al.*, 2005; “BARE room”). The inner dimensions of the booth were approximately 3.8 × 4.0 × 2.3 m (length × width × height). Seven visible loudspeakers were positioned on a horizontal arc with a radius of 1.5 meters centered on the participant, at azimuths of 0°, ±15°, ±30°, and ±90°. In Experiment 1, stimuli were only presented from the loudspeaker at 0°. Experiment 2 made use of all seven loudspeakers. Participants were asked to keep their heads still during the experimental blocks, but their head was not restrained.

Stimuli were generated on a Lenovo PC using matlab 2019 b (MathWorks, Inc., Natick, MA) at a sampling rate of 44 100 Hz, passed to a multichannel soundcard (MOTU 16 A, MOTU, Inc., Cambridge, MA) and a bank of power amplifiers (Crown Audio XTi 1002, Crown Audio, Los Angeles, CA) all located outside of the booth, and then delivered to the loudspeakers (Acoustic Research 215 PS, Acoustic Research, Cambridge, MA). Participants provided responses using a handheld backlit keypad.

### 2.4 Stimuli and procedures

The stimuli included environmental sounds and speech sounds interleaved randomly in 252-trial blocks. Following each stimulus presentation, the participant rated their perceived externalization using a continuous integer scale (10 = at the loudspeaker; 0 = inside the head; −10 = behind the head at an equal distance to the front loudspeaker). Note that while we have chosen to use the term “externalization ratings,” these can also be considered as near-field distance ratings (for a discussion, see Best *et al.*, 2020).

**Fig. 1.**
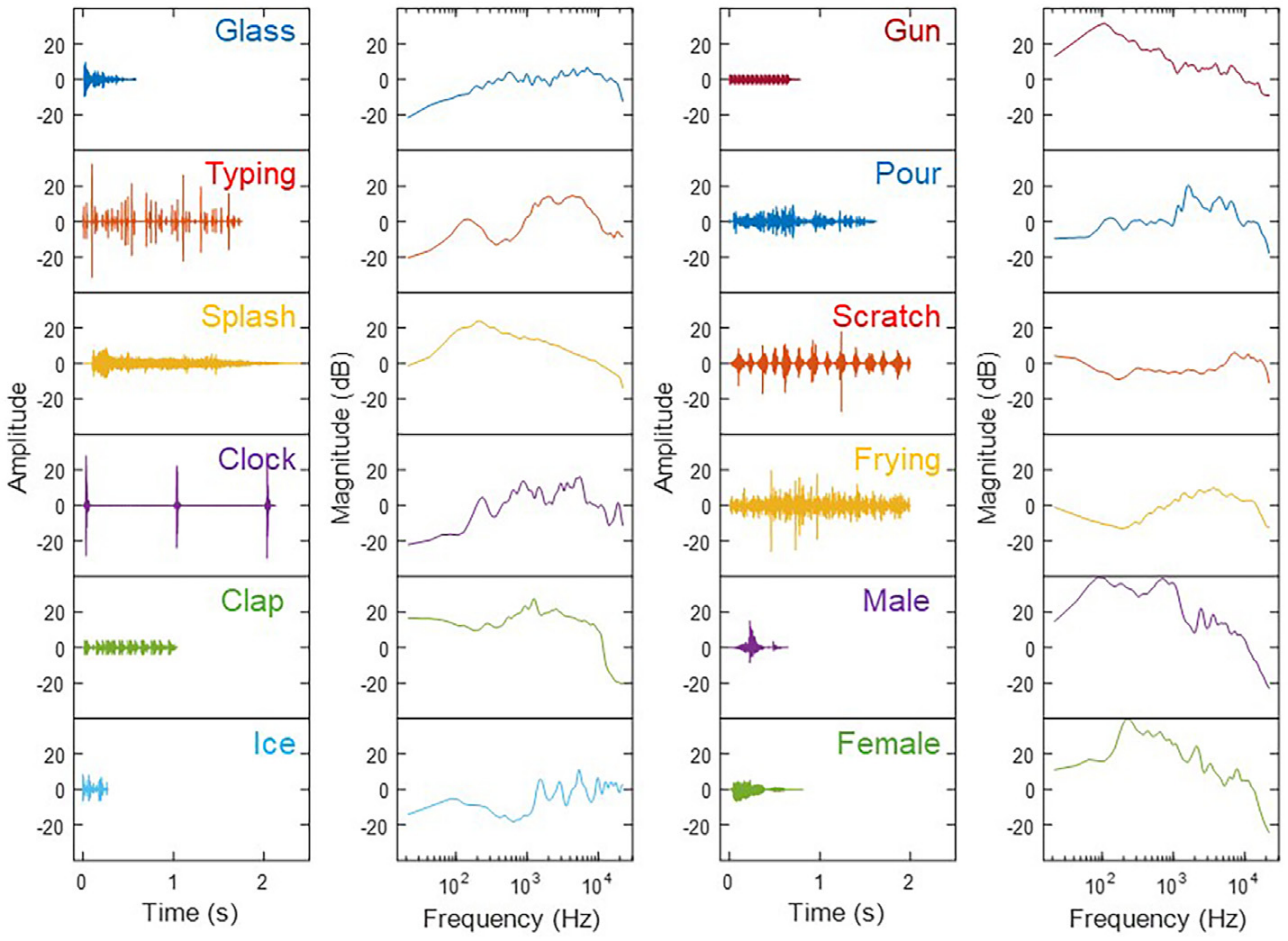
Waveforms and spectra of each of the tokens. The magnitude values for the spectra are normalized according to the mean value across frequency for that specific token. The male and female voice tokens shown are those from Experiment 2.

The environmental sounds were 10 tokens selected from corpora shared by Shafiro (2008), Gygi and Shafiro (2019), and Norman-Haignere *et al.* (2015). The tokens were selected somewhat arbitrarily, but were all broadband and represented a wide variety of temporal characteristics. Details about the environmental sounds are provided in Table 1 and Fig. 1. The speech sounds were monosyllabic words selected from a corpus recorded at Boston University (described in Kidd *et al.*, 2008; word options selected from those listed in Roverud *et al.*, 2020). The set included 16 words (eight adjectives and eight nouns) spoken by each of four talkers (two male and two female). Table 1 and Fig. 1 include details of the speech stimuli for comparison to the environmental sounds.

**Table 1.** Details of the sound tokens used. Parenthetical letters after each token description indicate the corpus the token was selected from (A = Gygi and Shafiro, 2019; B = Shafiro, 2008; C = Norman-Haignere *et al.*, 2015; D = Kidd *et al.*, 2008). Values for the male and female talkers in Experiment 1 represent averages across 32 words.

Token description	Shorthand name	Duration (s)	Crest factor	Spectral centroid (Hz)
Glass breaking (B)	Glass	0.59	9.68	9773
Typing on a keyboard (B)	Typing	1.76	32.2	4263
Water splashing (A)	Splash	2.40	8.73	2274
Clock ticking (B)	Clock	2.13	29.7	4507
Clapping/applause (B)	Clap	1.04	3.98	3299
Ice clinking in a glass (B)	Ice	0.28	8.34	9748
Machine gun fire (B)	Gun	0.78	2.63	1393
Water pouring into a glass (B)	Pour	1.62	9.19	4926
Scratching noise (C)	Scratch	2.00	27.1	9695
Oil frying in a frypan (C)	Frying	2.00	25.9	6313
Male talker (D)	Male	Exp 1 = 0.68, Exp 2 = 0.65	Exp 1 = 7.37, Exp 2 = 15	Exp 1 = 1658, Exp 2 = 1822
Female talker (D)	Female	Exp 1 = 0.64, Exp 2 = 0.81	Exp 1 = 5.49, Exp 2 = 6.87	Exp 1 = 1792, Exp 2 = 2129

In Experiment 1, each of the ten environmental tokens was presented twice at three presentation levels (50, 55, and 60 dB SPL) for a total of 60 environmental sounds per block, and each of the 64 speech tokens was presented once at the same three levels (192 speech tokens per block). After seven participants had completed Experiment 1, it was clear that they found the blocks monotonous, and we suspected that the lack of variability in the stimuli was an issue. Thus, the mix of stimuli was modified for Experiment 2 to include seven azimuths instead of one, and to achieve a better balance of environmental and speech sounds. In Experiment 2, each of the ten environmental tokens was presented once for each of the three levels and seven loudspeakers (210 environmental sounds per block). The speech tokens were reduced to one female talker speaking the word “bags” and one male talker speaking the word “hot” (preliminary analysis of the data from Experiment 1 indicated that these specific words yielded the largest HA effects). These two words were presented once for each of the three levels and seven loudspeakers (42 speech tokens per block).

Each participant completed one block of trials per HA condition (unaided, linear, compressive) in a random order before repeating each of the three conditions in a new randomized order. The exceptions to this were for the first two participants in Experiment 1, who only ran the unaided condition once at the end of the session.

## 3. Results

### 3.1 Externalization as a function of sound token

**Fig. 2.**
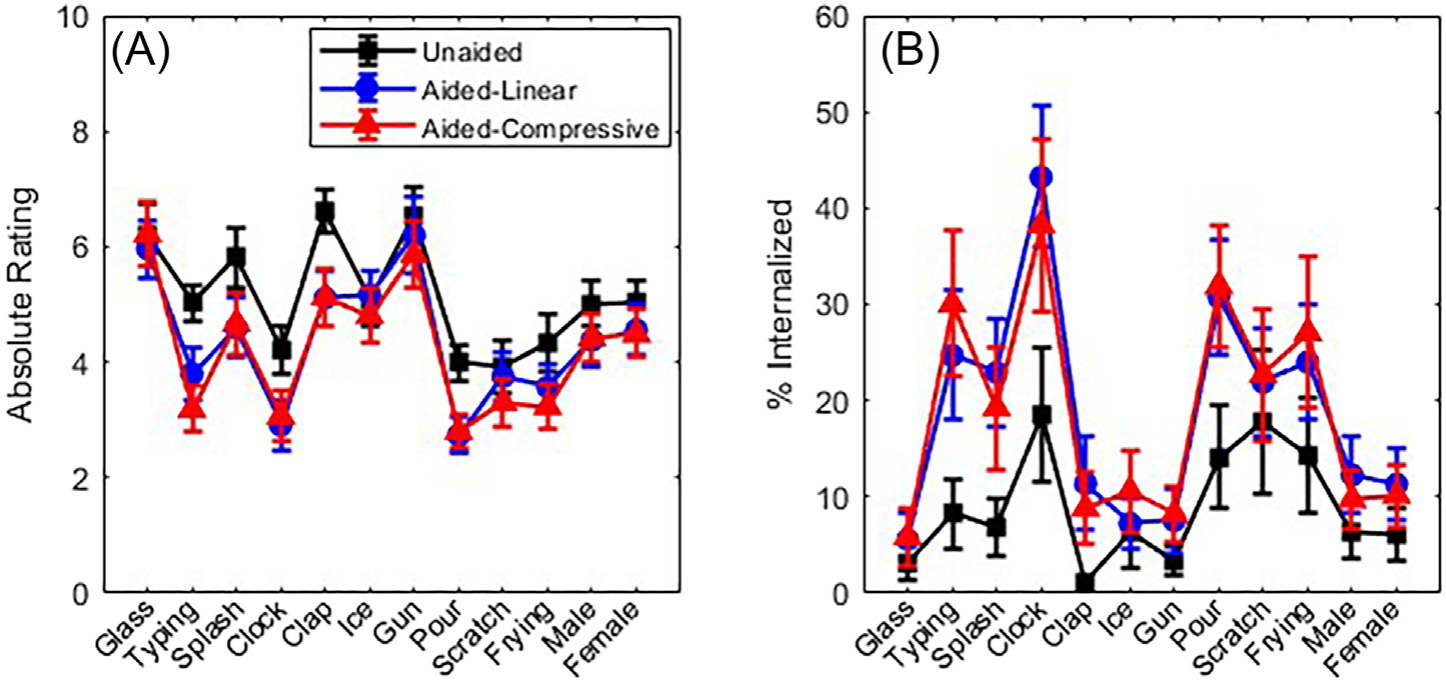
Absolute externalization ratings (A) and the percentage of internalized responses (B) as a function of sound token for each HA condition. Symbols and error bars show across-subject means and standard errors. Symbols within each HA condition are connected by lines to facilitate across-condition comparisons.

Although the response scale allowed for the possibility of front-back reversals, these were relatively rare and not of primary interest in the current experiment, so they are not considered further here. For the analyses that follow, externalization ratings represent absolute values (i.e., rear responses are flipped to the front). Figure 2(A) shows absolute externalization ratings for each HA condition as a function of sound token. These results are based on averages across level, azimuth, experiment, and participant. Figure 2(B) shows the percentage of internalized responses (defined as absolute ratings corresponding to 0 or 1, as in Best and Roverud, 2024) in the same format. For all three HA conditions, there are similar variations across tokens, with lower ratings and more internalized responses occurring for the “typing,” “clock,” and “pour” tokens. Also, ratings were generally higher (and internalized responses rarer) in the unaided condition than in the aided conditions. The differences between aided and unaided ratings were larger for some tokens than others. Differences between results for the linear and compressive HA conditions were generally small.

Trial-by-trial absolute externalization ratings were analyzed with a linear mixed-effects model using the *lmer* function in R. The independent variables included fixed effects of HA condition (coded categorically), presentation level (coded ordinally), sound token (coded categorically), the interaction of sound token × HA condition, and a random intercept for subject. Before settling on this model, we verified that including experiment (1 or 2) as a fixed effect did not significantly improve the model fit. We did not include a fixed effect of azimuth, given the uneven sampling of azimuth across the two experiments, and because this factor was not of primary interest. Sum contrast coding was used for categorical and ordinal variables. Significance testing for the fixed effects made use of the *anova* function (type III) in R. In a separate analysis, trial-by-trial data coded as 1 (internalized) or 0 (not internalized) were fitted with a generalized linear mixed model using the *glmer* function in R. This model was identical in structure to that applied to the rating data. Significance testing for the fixed effects made use of the *anova* function (type III) in R, which applies Wald Chi-square analysis of deviance tests for binomial data. Note that these two separate analyses were performed on the same data set and thus are not independent but provide complementary views of the data in terms of distance perception and internalization (see also Best and Roverud, 2024).

As shown in the top section of Table 2, all fixed effects (HA condition, level, token) and the interaction of HA condition × token were statistically significant for both ratings and internalized responses. Post hoc Tukey contrasts for significant effects were conducted with the *emmeans* function. Contrasts involving HA condition (not shown in table) revealed that both linear and compressive HAs yielded lower ratings and more internalized responses than unaided, but linear and compressive conditions did not differ from each other. All three presentation levels were significantly different from one another (not shown in table), with higher levels associated with lower ratings and more internalized responses than lower levels. The statistical significance of contrasts for the HA condition × token interaction is shown in the bottom section of Table 2. For most tokens, ratings were lower and internalization was more common with HAs than unaided, except for the “Glass,” “Ice,” and “Scratch” tokens, in which the effect of aiding was absent or inconsistent. There was no token for which the compressive and linear aided conditions differed significantly for both ratings and internalized responses.

**Table 2.** Statistical analysis of absolute ratings and internalized responses, considering the factors of HA condition, level, and token. Key model results and post hoc contrasts are shown in the top and bottom sections, respectively.

	Absolute ratings			Internalization		
	Type III ANOVA table with Satterthwaite's method			Analysis of deviance table (Type III Wald chisquare tests)		
	df (num/den)	*F* value	*p* value	df	Chi Sq	*p* value
**HA**	2/16583	257.85	*<0.001*	2	196.35	*<0.001*
**Level**	2/16583	2116.33	*<0.001*	2	951.09	*<0.001*
**Token**	11/16584	226.39	*<0.001*	11	499.18	*<0.001*
**HA x Token**	22/16583	8.99	*<0.001*	22	101.38	*<0.001*

	Post hoc contrasts for HA × Token interaction, *p* values					
	Absolute ratings			Internalization		
	Compressive - linear	Compressive - unaided	Linear - unaided	Compressive - linear	Compressive - unaided	Linear - unaided
**Glass**	*0.02*	0.88	*0.003*	0.43	0.37	*0.04*
**Typing**	*<0.001*	*<0.001*	*<0.001*	0.05	*<0.001*	*<0.001*
**Splash**	0.73	*<0.001*	*<0.001*	*0.048*	*<0.001*	*<0.001*
**Clock**	0.19	*<0.001*	*<0.001*	*0.01*	*<0.001*	*<0.001*
**Clap**	0.99	*<0.001*	*<0.001*	0.39	*<0.001*	*<0.001*
**Ice**	0.92	0.11	0.23	0.98	0.09	0.14
**Gun**	0.36	*<0.001*	*0.02*	0.98	*0.01*	*0.02*
**Pour**	0.89	*<0.001*	*<0.001*	0.97	*<0.001*	*<0.001*
**Scratch**	0.10	*<0.001*	0.06	0.71	0.10	*0.01*
**Frying**	*0.04*	*<0.001*	*<0.001*	0.65	*<0.001*	*<0.001*
**Male**	0.70	*<0.001*	*<0.001*	0.25	*<0.001*	*<0.001*
**Female**	0.96	*<0.001*	*<0.001*	0.91	*0.003*	*<0.001*

### 3.2 Predicting externalization using acoustic properties

To further examine the significant effects involving token, we extracted two key acoustical properties of each token (crest factor and spectral centroid; Table 1). Note that because the crest factor was taken as the peak level to root-mean-square (RMS) ratio, and stimuli were RMS-normalized, crest factor is equivalent to peak level. Crest factor and spectral centroid were not correlated across tokens (Pearson's *r* = 0.35, *p =* 0.22).

These acoustical measures were included in a second set of statistical models similar to those presented previously. The dependent variables were again absolute ratings and internalized responses. In this case, however, the fixed effects were HA condition, level, and *z*-scored crest factor and spectral centroid values. Given the lack of a significant difference between compressive and linear HA conditions found previously, HA condition was reduced to two levels for this analysis (unaided or aided). The two-way interactions between the HA condition and each of the acoustical measures were also included, as was a random intercept for the subject. HA condition and level were coded using sum contrast coding.

The results of these model fits are shown in the top section of Table 3. For ratings, all fixed effects and the two-way interactions of HA condition x crest factor and HA condition × spectral centroid were significant. For internalized responses, most fixed effects were significant (but not spectral centroid), as was the two-way interaction of HA condition × crest factor (but not the interaction of HA condition × spectral centroid). Regarding the post hoc contrasts of the significant main effects (not shown in the table), ratings were lower and internalization was more likely for the aided condition (compared to unaided), for higher presentation levels (compared to lower presentation levels), and for tokens with a higher crest factor (compared to lower). Additionally, ratings were lower for tokens with a lower spectral centroid (compared to higher). Contrasts for the interactions are shown in the bottom section of Table 3, with estimates indicating slopes relating crest factor or spectral centroid to predicted ratings and log-odds internalization for aided and unaided conditions. For both ratings and internalized responses, crest factor was a significant predictor for both aided and unaided conditions, but it was a significantly stronger predictor in the aided condition. For ratings, spectral centroid was a significant predictor for both aided and unaided conditions but was a significantly stronger predictor in the aided condition. For internalized responses, although the HA condition × spectral centroid interaction was not significant, the spectral centroid was significant for the aided condition. Overall, these results indicate that tokens with higher crest factors were more likely to produce breakdowns of externalization for both aided and unaided conditions, but especially when aided. Moreover, with HAs, an additional disruption to externalization was apparent for tokens with lower spectral centroids.

**Table 3.** Statistical analysis of absolute ratings and internalization using predictors of HA condition, level, z-scored crest factor, and z-scored spectral centroid. Key model results and post hoc contrasts are shown in the top and bottom sections, respectively.

	Absolute ratings			Internalization		
	Type III ANOVA table with Satterthwaite's method			Analysis of deviance table (Type III Wald Chisquare tests)		
	df (num/den)	*F* value	*p* value	df	Chi Sq	*p* value
**HA**	1/16 614	466.16	*<0.001*	1	221.49	*<0.001*
**Level**	2/16 613	1945.80	*<0.001*	2	927.53	*<0.001*
**Crest factor**	1/16 615	801.37	*<0.001*	1	177.77	*<0.001*
**Spectral centroid**	1/16 618	41.89	*<0.001*	1	2.95	0.09
**HA × Crest Factor**	1/16 613	45.08	*<0.001*	1	16.96	*<0.001*
**HA × Spectral Centroid**	1/16 613	10.76	*0.001*	1	0.16	0.69

	Post hoc contrasts for significant interactions					
	Absolute ratings			Internalization		
	Estimate	*t* ratio	*p* value	Estimate	*Z* ratio	*p* value
**Crest factor**						
** Aided**	−0.76	−30.36	*<0.001*	0.73	17.94	*<0.001*
** Unaided**	−0.47	−13.45	*<0.001*	0.39	5.42	*<0.001*
**Spectral centroid**						
** Aided**	0.21	8.42	*<0.001*	−0.09	−2.20	*0.03*
** Unaided**	0.07	2.08	*0.04*	−0.06	−0.76	0.44

## 4. Discussion

The primary aim of this study was to investigate whether certain everyday sounds are more prone to the disrupting effects of HAs on sound externalization. We replicated our previous findings (Best and Roverud, 2024) showing lower externalization ratings and a greater tendency for internalized responses for aided compared to unaided listening. We also extended this result beyond speech to a range of environmental sounds and found a significant effect of the specific token, with disruptions to externalization for some tokens but not others.

An analysis involving two acoustical properties of the different sound types—crest factor (or peak level) and spectral centroid—suggested that the crest factor may have contributed to the overall variations in externalization; higher crest factors were associated with weaker externalization for both unaided and aided conditions. The tokens with the highest crest factors included “Typing,” “Clock,” “Scratch,” and “Frying,” and these tokens were all rather poorly externalized. The relationship between crest factor and externalization was stronger with HAs, suggesting that HAs exacerbate this general effect. Spectral centroid, which describes the spectral region with the highest concentration of energy for these broadband sounds, was associated with additional variations in externalization with HAs; tokens with a lower frequency spectral centroid were more likely to be given lower (closer) ratings with HAs than without. Demonstrating this effect, the two tokens with the highest spectral centroids (“Glass,” “Ice”) were also the tokens that showed little difference between aided and unaided conditions. This result runs somewhat counter to the findings of Boyd (2014) and Wiggins and Seeber (2012), which implicated *high-frequency* sounds in breakdowns of externalization, but this may reflect the use of broadband (not narrowband) stimuli in the current study.

We tested both linear and compressive HAs, to understand if the temporal aspects of compressive gain application were important drivers of any observed HA effects. If the nonlinear effects of compression did indeed alter the stimulus waveforms, the alterations were apparently not sufficient to influence externalization ratings. Instead, our results suggest that the provision of either linear or compressive gain through these particular devices had a disruptive effect on the externalization of many everyday sounds.

Additional information regarding the importance of stimulus acoustics in sound externalization, with and without HAs, may be revealed in future studies using a broader sampling of sounds or systematic manipulations of different features of sounds. Specifically, it would be worth considering narrowband sounds, which are poorly externalized in general (Best *et al.*, 2020) and may be particularly susceptible to the effects of HAs (Boyd, 2014). Another interesting future direction would be to investigate a larger set of broadband sounds with different spectral profiles. Since HAs alter the spectrum of incoming sounds in various ways, their effects on externalization may vary with different sound source spectra. Our preliminary acoustic analysis suggested that spectral centroid may be relevant, but other characteristics may also play a role. For example, effects related to microphone position, which involve high-frequency spectral details, may be most apparent for stimuli with a relatively flat spectrum. Effects related to occluding domes, which tend to boost low frequencies, may be more noticeable for low-frequency dominant sounds such as speech. Another intriguing question is whether “unfamiliar” or “unexpected” sounds in the environment are more difficult to place accurately in extra-personal space. In addition, it is important for future work to extend these results to reverberant environments in which HAs may also interact with the acoustics of the room to affect externalization (e.g., Hassager *et al.*, 2017).

Finally, while our study was limited to listeners with normal hearing, different results may be expected from listeners with hearing loss who are long-term users of HAs. These listeners may have adapted to the specific disruptive effects of their devices (e.g., related to microphone position and dome type) and thus may show better externalization overall when listening aided. On the other hand, most HA users would have higher compression ratios than those used in the current study, and thus may show larger differences between linear and compressive amplification.

## 5. Conclusions

This study confirmed that, for listeners with normal hearing who are fit with low-gain HAs, sounds are perceived to be too close and more often inside the head relative to the natural listening situation without HAs. Our results suggest that the disruptive effect of HAs—and the robustness of externalization in general—may vary widely depending on the spectrotemporal characteristics of everyday sounds.

## Data Availability

The data that support the findings of this study are available from the authors upon request.

## References

[1] Akeroyd, M. A. (2010). “ The effect of hearing-aid compression on judgments of relative distance,” J. Acoust. Soc. Am. 127, 9–12. 10.1121/1.3268505 20058945 PMC3514978

[2] Akeroyd, M. A., and Whitmer, W. (2016). “ Spatial hearing and hearing aids,” in *Hearing Aids. Springer Handbook of Auditory Research*, edited by G. Popelka, B. C. J. Moore, R. Fay, and A. Popper ( Springer, Geneva, Switzerland).

[3] Best, V., Baumgartner, R., Lavandier, M., Majdak, P., and Kopčo, N. (2020). “ Sound externalization: A review of recent research,” Trends Hear. 24, 233121652094839. 10.1177/2331216520948390 PMC7488874 32914708

[4] Best, V., and Roverud, E. (2024). “ Externalization of speech when listening with hearing aids,” Trends Hear. 28, 23312165241229572. 10.1177/23312165241229572 38347733 PMC10865954

[5] Boyd, A. (2014). “ Experimental investigations of auditory externalization and the application of head-movement information to hearing-aid signal processing,” Ph.D. thesis, University of Strathclyde, Glasgow, Scotland.

[6] Dillon, H. (2012). *Hearing Aids* ( Boomerang Press, Turramurra, Australia).

[7] Gygi, B. M., and Shafiro, V. (2019). “Database of Environmental Sounds for Research Activities (DESRA)—Sound files and metadata,” Zenodo. 10.5281/zenodo.2622626

[8] Hartmann, W. M. (2021). “ Localization and lateralization of sound,” in *Binaural Hearing*, edited by R. Y. Litovsky, M. J. Goupell, R. R. Fay, and A. N. Popper ( Springer, Cham).

[9] Hassager, H. G., Wiinberg, A., and Dau, T. (2017). “ Effects of hearing-aid dynamic range compression on spatial perception in a reverberant environment,” J. Acoust. Soc. Am. 141(4), 2556–2568. 10.1121/1.4979783 28464692

[10] Kidd, G., Jr., Best, V., and Mason, C. R. (2008). “ Listening to every other word: Examining the strength of linkage variables in forming streams of speech,” J. Acoust. Soc. Am. 124, 3793–3802. 10.1121/1.2998980 19206805 PMC2676624

[11] Kidd, G., Jr., Mason, C. R., Brughera, A., and Hartmann, W. M. (2005). “ The role of reverberation in release from masking due to spatial separation of sources for speech identification,” Acta Acust. United Acust. 91, 526–536.

[12] Noble, W., and Gatehouse, S. (2006). “ Effects of bilateral versus unilateral hearing aid fitting on abilities measured by the speech, spatial, and qualities of hearing scale (SSQ),” Int. J. Audiol. 45, 172–181. 10.1080/14992020500376933 16579492

[13] Norman-Haignere, S., Kanwisher, N. G., and McDermott, J. H. (2015). “ Distinct cortical pathways for music and speech revealed by hypothesis-free voxel decomposition,” Neuron 88(6), 1281–1296. 10.1016/j.neuron.2015.11.035 26687225 PMC4740977

[14] Roverud, E., Bradlow, A. R., and Kidd, G., Jr. (2020). “ Examining the sentence superiority effect for sentences presented and reported in forwards or backwards order,” Appl. Psycholing. 41(2), 381–400. 10.1017/S014271642000003X PMC8191368 34121781

[15] Shafiro, V. (2008). “ Development of a large-item environmental sound test and the effects of short-term training with spectrally-degraded stimuli,” Ear Hear 29(5), 775–790. 10.1097/AUD.0b013e31817e08ea 18596641

[16] Stone, M. A., and Moore, B. C. J. (1992). “ Syllabic compression: Effective compression ratios for signals modulated at different rates,” Br. J. Audiol. 26, 351–361. 10.3109/03005369209076659 1292819

[17] Wiggins, I. M., and Seeber, B. U. (2012). “ Effects of dynamic-range compression on the spatial attributes of sounds in normal-hearing listeners,” Ear Hear. 33, 399–410. 10.1097/AUD.0b013e31823d78fd 22246139

[18] Zahorik, P., Brungart, D. S., and Bronkhorst, A. W. (2005). “ Auditory distance perception in humans: A summary of past and present research,” Acta. Acust. united Ac. 91, 409–420.

